# Impact of Depression on Health Care Utilization and Costs among Multimorbid Patients – Results from the MultiCare Cohort Study

**DOI:** 10.1371/journal.pone.0091973

**Published:** 2014-03-17

**Authors:** Jens-Oliver Bock, Melanie Luppa, Christian Brettschneider, Steffi Riedel-Heller, Horst Bickel, Angela Fuchs, Jochen Gensichen, Wolfgang Maier, Karola Mergenthal, Ingmar Schäfer, Gerhard Schön, Siegfried Weyerer, Birgitt Wiese, Hendrik van den Bussche, Martin Scherer, Hans-Helmut König

**Affiliations:** 1 Department of Health Economics and Health Services Research, Hamburg Center for Health Economics, University Medical Center Hamburg-Eppendorf, Hamburg, Germany; 2 Institute of Social Medicine, Occupational Health and Public Health, University of Leipzig, Leipzig, Germany; 3 Department of Psychiatry, Technical University of Munich, Munich, Germany; 4 Department of General Practice, University of Düsseldorf Medical Center, Düsseldorf, Germany; 5 Department of General Practice, Jena University Hospital, Jena, Germany; 6 Department of Psychiatry and Psychotherapy, University of Bonn, Bonn, Germany; 7 Institute of General Practice, Goethe-University Frankfurt am Main, Frankfurt am Main, Germany; 8 Department of Primary Medical Care, University Medical Center Hamburg-Eppendorf, Hamburg, Germany; 9 Department of Medical Biometry and Epidemiology, University Medical Center Hamburg-Eppendorf, Hamburg, Germany; 10 Central Institute of Mental Health, Medical Faculty Mannheim/Heidelberg University, Mannheim, Germany; 11 Institute for Biometry, Hannover Medical School, Hannover, Germany; University of Adelaide, Australia

## Abstract

**Objective:**

The objective of this study was to describe and analyze the effects of depression on health care utilization and costs in a sample of multimorbid elderly patients.

**Method:**

This cross-sectional analysis used data of a prospective cohort study, consisting of 1,050 randomly selected multimorbid primary care patients aged 65 to 85 years. Depression was defined as a score of six points or more on the Geriatric Depression Scale (GDS-15). Subjects passed a geriatric assessment, including a questionnaire for health care utilization. The impact of depression on health care costs was analyzed using multiple linear regression models. A societal perspective was adopted.

**Results:**

Prevalence of depression was 10.7%. Mean total costs per six-month period were €8,144 (95% CI: €6,199-€10,090) in patients with depression as compared to €3,137 (95% CI: €2,735-€3,538; p<0.001) in patients without depression. The positive association between depression and total costs persisted after controlling for socio-economic variables, functional status and level of multimorbidity. In particular, multiple regression analyses showed a significant positive association between depression and pharmaceutical costs.

**Conclusion:**

Among multimorbid elderly patients, depression was associated with significantly higher health care utilization and costs. The effect of depression on costs was even greater than reported by previous studies conducted in less morbid patients.

## Introduction

Depression in old age is common [1], causing substantial negative consequences for the affected individual such as decreased quality of life, functional impairment and marked disability [2]. As a result of demographic aging in many industrialized countries, depression in old age will become even more relevant in future. Several studies have shown that depression in old age is associated with higher resource utilization and costs [3].

The growing number of old people resulting from demographic aging is accompanied by an increasing number of patients living with multiple chronic conditions, also referred to as multimorbidity. Multimorbidity is often defined as the co-occurrence of two or more (chronic) illnesses in one person without reference to one index disease [4] and is frequent in old age [5]. Many studies have shown that multimorbidity is associated with higher health care utilization and costs [6]. Therefore, the growing number of old people with multiple chronic conditions will also become more important from a health economic point of view.

As a consequence, both depression and multimorbidity pose a great burden on health care systems and societies. It has been shown that the prevalence of depression increases with the number of chronic conditions [7]. Yet, the impact of depression on health care utilization and costs among multimorbid patients has not been investigated so far.

The objective of this study was to describe and analyze the effects of depression on health care utilization and costs in a sample of multimorbid patients. In this bottom-up cost-of-illness study a societal perspective was adopted and incremental costs of depressed compared to non-depressed patients were calculated.

## Materials and Methods

### Ethics statement

The study was approved by the Ethics Committee of the Medical Association of Hamburg (Approval-No. 2881) and conducted according to the principles expressed in the declaration of Helsinki. Written informed consent was obtained previous to inclusion into the study.

### Sample

**Figure 1:**
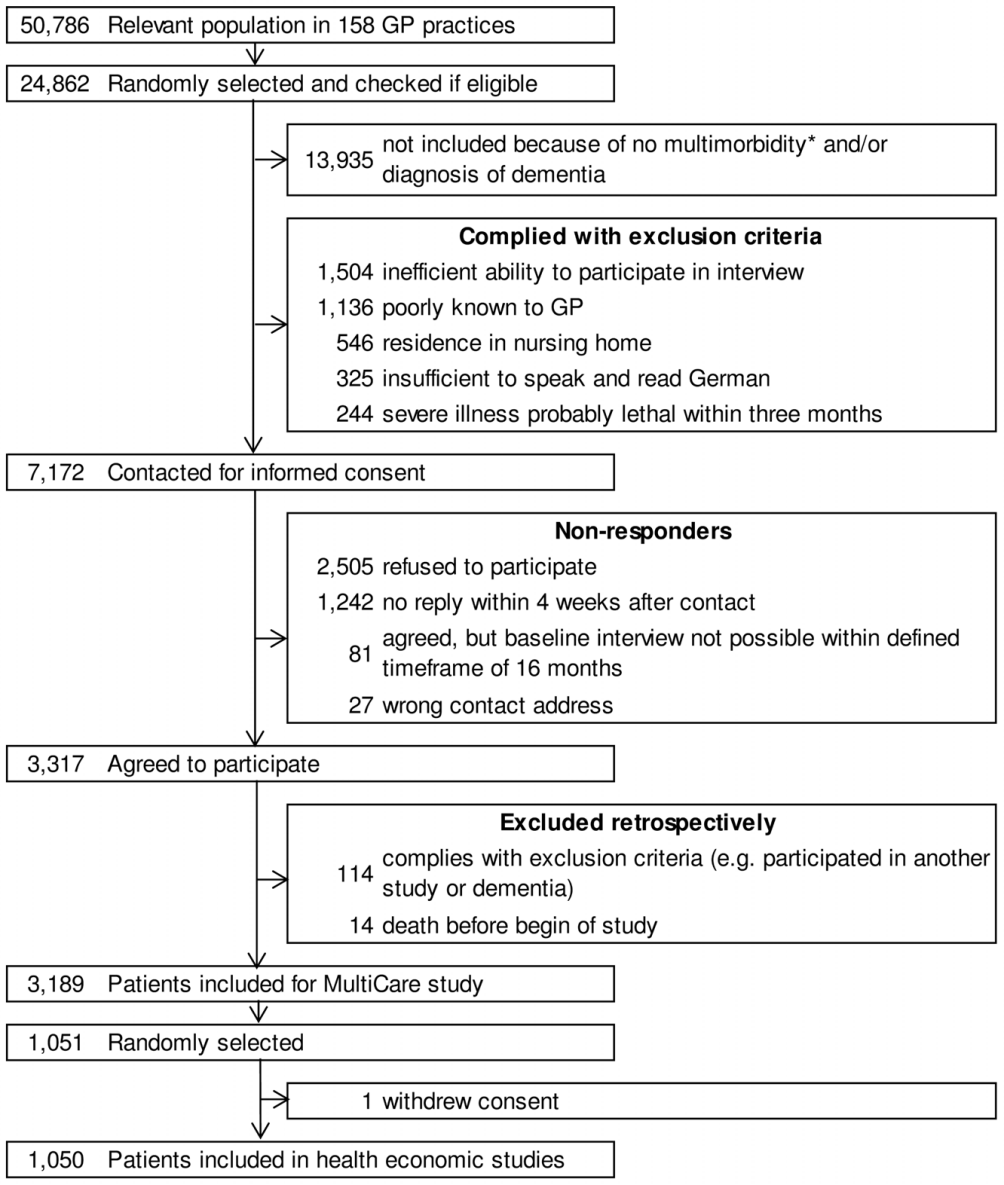
Sample selection. Figure adapted from [8]. *Mutimorbidity was defined as at least 3 out of 29 ICD-10-based diagnosis groups.

This cross-sectional analysis used baseline data of the MultiCare Cohort Study - a multicentre, prospective, observational cohort study of patients aged 65 to 85 years suffering from multiple chronic conditions. Patients were recruited via 158 GP practices in eight different German cities. From 50,786 patients of the participating GPs, 24,862 were randomly selected. Of these 24,862, 13,935 without multimorbidity, defined as co-occurrence of three or more chronic conditions from a list of 29 diseases, were excluded. Furthermore, 3,755 patients were excluded for the following reasons: no regular patient of the participating practice, unable to participate in interviews (especially blindness and deafness), not able to speak and read German, residence in a nursing home, severe illness probably lethal within three months according to the GP, insufficient ability to consent (especially dementia) and participation in other studies. The resulting 7,172 people were contacted for informed consent and 3,317 of them agreed to participate. 128 were excluded retrospectively (e.g. for the diagnosis of dementia or death before the start of the study) resulting in 3,189 participants in the study. All participants provided comprehensive self-reported data about socio-economic, health and functional status collected by various standardized questionnaires. Additionally, clinical information was obtained from their GPs. Supplemental information on health care utilization was collected for 1,051 randomly selected participants. As one individual withdrew from the study before starting the standardized health economic questionnaire, the following analyses are based on a subsample of 1,050 randomly selected participants. Recruitment and baseline interviews took place between July 2008 and October 2009. Further information about the MultiCare Cohort Study has been reported elsewhere [5]. The selection of the study sample is illustrated in Figure 1.

### Health care utilization and unit costs

Data on health care utilization were collected via a patient questionnaire (previously used, e.g. by [9], [10], [11]) which recorded the resource consumption in seven health care sectors: Inpatient care, outpatient physician services, outpatient non-physician services, pharmaceuticals, medical supplies and dental prostheses, formal nursing care and informal nursing care. Resource utilization was recorded retrospectively for a period of three months, except for inpatient and nursing home care where the period was six months. In order to monetarily value the recorded resource units, e.g. physician contacts or hospital days, each service or good was valued with corresponding unit costs. Table 1 gives an overview about these unit costs. All units were valued in 2009 prices. If corresponding unit costs were only available for another date, the unit costs were in- or deflated using the consumer price index [12]. All costs were calculated for a six-month period, multiplying resource utilization by the factor two in sectors which were recorded for a three-month period.

**Table 1:** Recorded resources and source of monetary valuation, by health care sector.

Sector	Resources	Units	Source for monetary valuation
Inpatient treatment	Stays in general hospitals, specialized psychiatric and neurological hospitals or rehabilitation clinics (including day-patient treatment)	Days in hospital	Per diem costs by type (Federal Statistical Office, German Hospital Federation, Statutory Pension Insurance Fund; [13]–[16])
Outpatient phy-sician services	Treatment by GPs, specialists and outpatient clinics	Number of contacts	Calculated costs per contact, by specialization [17]
Outpatient non-physician services	E.g., physiotherapy, massage, occupational therapy, speech therapy	Number of contacts	Reimbursement schedules (Statutory health insurance funds; [18]–[20]), calculated costs per contact [17], by type
Medical supplies and dental prostheses	E.g., walkers, incontinence pads, hearing aids, surgical stockings; bridge, crown	Quantity	Reimbursement schedules (Statutory health insurance funds, Federal Association of Panel Dentists; [21], [22]), calculated costs per item [17], by type
Pharmaceuticals	Specific products (including trade name, drug code, package size, pharmaceutical form, dosage)	Quantity	Pharmacy retail prices (Rote Liste 2008; [23])
Nursing home care	Nursing home home stays (residential and day care)	Days	Calculated costs of care per day (Federal Statistical Office [24]), by type
Professional nursing care	Care and assistance provided by professional nursing services and other paid help, differentiated by type (e.g., basic care, assistance with cleaning, shopping, financial matters etc.) and limited to care or assistance required due to illness or age	Hours	Hourly gross wage rate plus non-wage labor costs for employees in the domain of care and assistance for the elderly or handicapped (Federal Statistical Office; [25], [26])
Informal care	Care and assistance provided by family or friends, differentiated by type and limited to care or assistance required due to illness or age	Hours	Replacement cost method: Hourly gross wage rate plus non-wage labor costs for employees in the domain of care and assistance for the elderly or handicapped (Federal Statistical Office; [25], [26])

Indirect costs were not considered, since the sample only consisted of participants above the statutory retirement age of 65 years. Thus, we followed the simplifying assumption of no societal productivity losses originating from these considered elderly.

### Depressive symptoms

Depressive symptoms were measured using a short form of the Geriatric Depression Screening Scale, the GDS-15 [27], [28], a self-rating scale with a score ranging from 0 to 15 points. Following recommendations for the German health care context [29], [30], a score of six points or more on this scale was defined as being depressed, in the following also referred to as “depression”.

### Independent predictor variables

Andersen and Newman [31] analyzed individual determinants of health care utilization and divided corresponding factors in three groups: predisposing characteristics, enabling resources and illness level. The selection and presentation of independent variables follows this structure.

Predisposing characteristics are socio-cultural predictors of health care utilization that are not related to an individual's specific health status. As such, we used general socio-demographic variables like age, gender and marital status (married, married but separate, single, divorced, widowed) as well as the educational level, operationalized by the CASMIN classification [32]. We used a variant of the international CASMIN classification that consists of three classes of educational level (low, middle, high). Social support by family, friends, neighbours, etc. was measured using the standardized questionnaire F-SOZU K-14 [33], which is a short form of the F-SOZU [34]. The F-SOZU K-14 summarizes the 14 collected items to one index ranging from 0 (bad social support) to 5 (optimal social support).

Enabling resources cover relevant logistical factors that determine – besides the predisposing socio-cultural factors – health care utilization. As such factors the type of health insurance (Statutory Health Insurance [SHI] or Private Health Insurance [PHI]) was considered. Furthermore, the regular monthly net income of the household from all sources was used. We applied the so-called modified OECD scale that divides the household income by the number of individuals living in the household. The first person was given the weight ‘1’, any further person was weighted by ‘0.5’ or with ‘0.3’ if younger than 15 years in order to adjust for synergy effects of larger households.

The illness level was operationalized, on the one hand, as functional status measured by the Barthel index [35]. This index ranges from 0 (worst) to 100 (best) and covers ten dimensions of activities of daily living, e.g. toilet use, feeding, walking, dressing etc. If such an activity is not possible at all without help of another person, this category is assessed with zero points. The index is created by the sum of each category's points.

On the other hand, the illness level was assessed by GP's diagnoses of 46 different chronic conditions. The process of the selection of these 46 chronic conditions has been reported elsewhere [36]. The severity of each respective chronic condition was assessed by the GP by giving 1 to 4 points to each existing chronic condition. A weighted count score for multimorbidity was created, consisting of the amount of severity points. As ‘depression’ was one of the initial chronic diseases, the weighted count score for comorbidity (multimorbidity apart from depression) for the following analyses only consists of the remaining 45 diseases and the described count score of the respective severities.

### Missing values and statistical analyses

Missing values in the above presented independent variables were imputed using the hot-deck-method [37]. Further details concerning the applied imputation method can be found elsewhere [8]. Missing values in variables documenting the resource consumption were imputed using conditional means. In the questionnaire, all resource utilization items were introduced by the question whether this resource had been used or not. If the participant stated ‘yes’, but then left out the corresponding quantity, the missing value was imputed by the means of the corresponding residual values.

Income data were missing in 12.7% of cases. The severity of the chronic illnesses could not be calculated in 4.5% of the total cases due to missing values. In all other categories the percentage of missing values did not exceed 0.5%.

All statistical analyses were performed using Stata 11 SE. The level of significance was set to α = 0.05. Differences in means were analyzed using the Student's t-test, differences in proportions were analyzed using the Chi-square test. Multivariate analysis of the impact of depression on health care costs was analyzed using multiple linear regression models (ordinary least squares). As health care costs were skewed to the right, standard errors were estimated using non-parametric bootstrapping (2,000 replications).

From the societal perspective the inclusion of informal care is essential [38]; however, as there is no consensus on how to value informal care [39], we performed a sensitivity analysis for total costs excluding cost of informal care, as recommend in the literature [40].

## Results

### Sample characteristics

Among the 1,050 participants the prevalence of depression (GDS-score≥6) was 10.7% (112 participants). The mean age of the sample was 74.4 (SD: 5.2) years, with depressed participants being slightly older than the non-depressed group. 58.7% of participants were female and 57.0% were married. Both, in gender and marital status, there were no statistically significant differences between participants with depression and the non-depressed group. Yet, compared the non-depressed participants, participants with depression received less social support (F-SozU), had less income, a worse average functional status (Barthel-score) and a higher comorbidity level (weighted count score for comorbidity), with each difference being statistically significant. Table 2 summarizes the sample characteristics.

**Table 2:** Socio-demographic and health-related sample characteristics.

Characteristics	All		Non-depressed		Depressed		p- value^b^	Missings
			(GDS^a^<6)		(GDS^a^≥6)			(%)
Age: mean (SD)	74.4	(5.2)	74.3	(5.1)	75.1	(5.6)	0.143	0.0
Sex	1,050	100%	938	100%	112	100%	0.283	0.0
- male	434	41%	393	42%	41	37%		
- female	616	59%	545	58%	71	63%		
Marital status	1,050	100%	937	100%	112	100%	0.139	0.1
- married	598	57%	547	58%	51	46%		
- married but seperate	26	2%	23	2%	3	3%		
- single	68	6%	58	6%	10	9%		
- divorced	68	6%	60	6%	9	8%		
- widowed	289	28%	250	27%	39	35%		
Social network (F-SozU K14):	4.1	(0.7)	4.2	(0.6)	3.5	(0.9)	0.000	0.4
mean (SD)								
Educational level	1,050	100%	938	100%	112	100%	0.070	0.0
- low	649	62%	576	61%	73	65%		
- middle	297	28%	274	29%	23	21%		
- high	104	10%	88	9%	16	14%		
Income in Euro: mean (SD)	1,440	(737)	1,462	(751)	1,253	(579)	0.004	12.7
Health insurance	1,050	100%	938	100%	112	100%	0.654	0.0
- statutory	998	95%	891	95%	107	96%		
- private	45	4%	40	4%	5	4%		
- others	7	1%	7	1%	0	0%		
Barthel index score: mean (SD)	97.9	(6.9)	98.3	(6.3)	94.3	(10.3)	0.000	0.3
Comorbidity score: mean (SD)	11.0	(5.8)	10.8	(5.1)	12.8	(5.9)	0.000	4.5

a GDS: Geriatric Depression Scale;

b differences in proportions: χ^2^-test; differences in means: t-test; SD: standard deviation.

### Descriptive analyses: resource utilization and mean costs

Table 3 gives an overview about the resource utilization and the corresponding costs per six months for participants with and without depression and for the entire sample by health care sector.

**Table 3:** Resource utilization and costs in Euro for 6-month period, total and by health care sector.

Health care sector	Proportion of users		Costs in Euro						
	Non-depressed	Depressed	All		Non-depressed		Depressed		p-value^b^
						
	(GDS^a^<6)	(GDS^a^≥6)			(GDS^a^<6)		(GDS^a^≥6)		
	(%)	(%)	Mean	(SD)	Mean	(SD)	Mean	(SD)	
Inpatient treatment	19.08^c^	33.93^c^	1,096	(4,030)	974	(3,931)	2,125	(4,669)	0.004
Outpatient physician services	98.40^d^	99.11^d^	418	(846)	402	(880)	553	(458)	0.076
Outpatient non-physician services	48.29^d^	57.14^d^	141	(245)	135	(232)	192	(332)	0.022
Medical supplies	35.61^d^	52.68^d^	135	(420)	130	(423)	180	(399)	0.236
Pharmaceuticals	99.57^d^	98.21^d^	590	(753)	546	(702)	958	(1,021)	0.000
Formal nursing care	8.85^c^	21.43^c^	105	(483)	80	(387)	316	(945)	0.000
Informal care	15.78^c^	41.96^c^	1,185	(4,745)	870	(4,006)	3,821	(8,335)	0.000
Total costs			3,671	(6,996)	3,137	(6,271)	8,145	(10,391)	0.000

a GDS: Geriatric Depression Scale;

b differences in means of costs per respondent between GDS<6 and GDS≥6: t-test;

c proportion of users in 6-month period;

d proportion of users in 3-month period.

During the six months preceding the interview, one third of the participants with depression had been admitted to a hospital as opposed to only 19% of the non-depressed group. Consequently, mean costs of inpatient care per six months were more than twice as high in participants with depression (€2,125; 95% CI: €1,251–€2,999) compared to those without depression (€973; 95% CI: €722–€1,226; p<0.01).

Most of the remaining formal health care costs were due to pharmaceuticals with almost all participants having used at least one pharmaceutical. However, the participants with depression caused significantly higher mean costs per six months (€958; 95% CI: €767-€1,149) than the non-depressed group (€546; 95% CI: €501–€591; p<0.001).

Virtually all participants used outpatient physician services. However, mean costs per six months tended to be higher in participants with depression (€553; 95% CI: €467–€638) than in the non-depressed group (€402; 95% CI: €346–€459), with this difference being close to the level of statistical significance (p = 0.076).

Outpatient non-physician services were used by 57% of the participants with depression as compared to 48% of the non-depressed group during three months preceding the interview. The respective mean costs (extrapolated to a six months period) were significantly higher among the participants with depression (€192; 95% CI: €129–€253) than the non-depressed group (€135; 95% CI: €121–€150; p = 0.022). While medical supplies were used by 53% of participants with depression compared to only 36% of the non-depressed group during 3 months, the respective difference in mean costs was not statistically significant.

Formal nursing care was used by 21% of the participants with depression compared to only 9% of the non-depressed group during the six months preceding the interview with the respective mean costs being four times higher in participants with depression (€316; 95% CI: €139–€493 vs. €80; 95% CI: €55–€105; p<0.001). Furthermore, 42% of participants with depression received informal care compared to only 16% of the non-depressive group. Consequently, mean costs of informal care per six months monetarily valued by the replacement cost methods (see Table 1) were four times higher in participants with depression (€3,821; 95% CI: €2,261–€5,382) than in the non-depressed group (€870; 95% CI: €614–€1,127; p<0.001).

Summing up all sector-specific costs resulted in mean total costs per six months of €8,144 (95% CI: €6,199–€10,090) in participants with depression compared to only €3,137 (95% CI: €2,735–€3,538) in the non-depressed group (p<0.001).

Mental health service use contributed little to the difference in service use and costs between participants with depression and the non-depressed group. Of the participants with depression, only 6 (5.4%) had seen an outpatient psychiatrist and 5 (4.5%) an outpatient psychotherapist during the 3 months preceding the interview as compared to 7 (0.7%) and 6 (0.6%), respectively, of the non-depressed group. As a result, in participants with depression mean costs per six months were €3.30 for outpatient psychiatrist treatment and €11.33 for outpatient psychotherapist treatment compared to €0.36 and €2.37, respectively, in the non-depressed group. Only one participant with depression and two of the non-depressed group were admitted to a psychiatric hospital ward, increasing mean total costs per six months in the group of depressed participants by only €21 and €6 for the non-depressed group respectively.

### Multivariate analyses

Table 4 shows the results of multiple regression analyses for each considered health care sector and in total, with the respective costs (six months) as dependent variable. It shows that depression increased total costs per six months by €2,936 (95% CI: €1,023–€4,850; p<0.01). Among sector-specific costs, a significant impact of depression could be found on costs of pharmaceuticals which were increased by €327 (95% CI: €133–€522; p<0.001). In all other sector-specific analyses, the regression coefficient for depression was positive but not statistically significant. Besides depression, in particular the illness level measured by the Barthel index and the comorbidity score had a significant impact on total costs as well as various sector-specific costs. The adjusted coefficient of determination (R^2^ adjusted) varied among health care sectors, ranging from about 0.40 (medical supplies) to 0.01 (informal care). For total costs, this coefficient was 0.33. Without the covariate ‘depression’, the adjusted R^2^ of the regression model was 0.32, indicating that depression contributes relatively little to the explanation of the variance.

**Table 4:** Multiple regression analyses with six-month costs in Euro used as dependent variable, total an by health care sector.

Independent variables	Total	Inpatient	Physi-cian	Non-Phy-sician	Medical Supplies	Pharma-ceuticals	Nursing Care	Informal Care
								
	b (SE)	b (SE)	b (SE)	b (SE)	b (SE)	b (SE)	b (SE)	b (SE)
Depressed, GDS^a^≥6	2,936.1**	884.4	88.1	38.0	46.0	327.4***	132.2	1,420.1
(ref. GDS<6)	(976.3)	(515.8)	(93.4)	(29.7)	(42.0)	(99.1)	(86.2)	(758.3)
Marital status (ref. married)								
- married but separate	−367.0	485.6	33.7	30.8	−27.7	7.3	38.7	−935.4
	(864.4)	(1021.9)	(71.1)	(55.0)	(29.8)	(102.4)	(66.5)	(556.6)
- single	−500.2	159.1	51.4	17.8	118.9	30.7	27.7	−905.8
	(607.1)	(320.5)	(57.4)	(33.5)	(68.0)	(91.4)	(42.3)	(410.9)
- divorced	−202.5	134.5	120.1	−24.9	93.5	−23.2	1.0	−503.5
	(484.5)	(334.8)	(69.1)	(22.5)	(97.8)	(80.0)	(27.7)	(303.0)
- widowed	−141.4	197.4	51.3	−12.3	−36.2	−67.9	120.2**	−394.0
	(440.4)	(256.6)	(71.5)	(17.5)	(27.1)	(42.9)	(42.0)	(321.5)
Social network (F-SozU)	424.7	218.2	−26.9	6.4	28.3	−21.4	−18.3	238.4
(centered)	(317.9)	(224.4)	(52.5)	(11.1)	(19.4)	(34.4)	(29.3)	(191.9)
Income	−21.2	243.0	−22.0	32.3**	−10.3	32.1	30.1	−326.5
(centered)	(212.1)	(166.2)	(39.4)	(11.9)	(11.5)	(49.8)	(19.9)	(144.5)
Age	−10.3	−17.6	−1.0	−3.3*	5.1	−6.5	10.9***	2.0
(centered)	(36.6)	(22.1)	(3.0)	(1.6)	(2.4)	(4.6)	(2.8)	(27.9)
Female	−128.3	−298.1	31.6	69.9***	22.4	−38.8	37.3	47.4
(ref. male)	(374.5)	(255.7)	(35.0)	(15.4)	(29.0)	(44.7)	(28.6)	(234.5)
Educational level (ref. low)								
- middle	−255.0	−231.8	13.1	44.2*	−30.0	15.8	6.8	−73.0
	(329.0)	(189.8)	(33.4)	(17.5)	(28.5)	(52.9)	(26.2)	(250.9)
- high	−644.3	−317.8	32.7	15.8	−67.4*	−47.4	93.6	−353.8
	(443.8)	(316.0)	(43.5)	(28.3)	(33.6)	(75.1)	(65.0)	(315.2)
Private health insurance^b^	1,015.6	619.5	54.8	139.2*	34.6	31.1	−109.5	245.8
(ref. statutory)	(701.4)	(595.1)	(61.5)	(63.5)	(53.4)	(83.7)	(45.0)	(520.1)
Barthel index score	−519.7***	−63.4	−0.5	−7.4***	−3.8	−11.4**	−15.9**	−417.3***
(centered)	(68.9)	(38.5)	(3.2)	(2.1)	(2.2)	(3.9)	(5.9)	(62.0)
Comorbidity score	167.1**	109.1	14.7*	1.5	−0.4	24.4***	4.8	13.1
(centered)	(64.3)	(61.9)	(6.3)	(1.2)	(2.7)	(3.9)	(2.7)	(19.6)
Constant	3,660.2***	1,147.5***	354.8***	78.3***	127.4***	593.5***	28.8	1,329.9***
	(383.5)	(313.0)	(23.1)	(12.7)	(28.1)	(42.7)	(18.8)	(200.2)
R^2^ (adjusted)	0.332	0.035	0.001	0.090	0.011	0.063	0.116	0.404
N	1,050	1,050	1,050	1,050	1,050	1,050	1,050	1,050

*** p<0.001; ** p<0.01; * p<0.05; SE: bootstrapped standard error (2,000 replications); ^a^GDS: Geriatric Depression Scale; ^b^Private health insurance including others.

When excluding costs of informal care in the sensitivity analysis, depression still had a significant positive influence on total costs, increasing them by about €1,516 (95% CI: €336–€2,696; p<0.05). The adjusted R^2^, however, was lower in this scenario and amounted to only 0.074.

## Discussion

### Main findings

The aim of this study was to describe and analyze the impact of depression on health care utilization and associated costs among multimorbid elderly. Excess costs of patients with depression were calculated from the societal perspective for the inpatient, outpatient physician and non-physician sectors, medical supplies including dental prostheses, pharmaceuticals as well as formal and informal care.

The analyses showed higher costs for each considered health care sector for patients with depression. In total, mean costs per patient with depression were €8,144 in a six-month period as compared to €3,137 for patients without depression. Thus, costs for patients with depression exceeded costs of those without by the factor 2.6. As participants with depression had a significantly worse functional status (measured by the Barthel index) and suffered from more severe comorbidity, multiple regression analyses were conducted for each health care sector in order to control for these factors. These analyses showed a significant positive association between depression and pharmaceutical costs as well as total costs. After controlling for socio-economic variables, functional status and comorbidity, depression still increased total costs by €2,936.

### Hypotheses on causes

Depression could per se lead to higher resource consumption by affected patients due to depression-specific treatment. Thus, higher utilization of mental health services and pharmaceuticals could indicate treatment of depression. However, in our study mental health services were only rarely used by patients with depression and contributed comparatively little to the sum of total costs. This goes in line with other studies which also showed that higher costs of elderly with depression can only in part be explained by utilization of resources directly related to the treatment of depression [41], [42], [43].

Furthermore, specific characteristics of depression could possibly lead to higher health care utilization. Patients with depression tend to worry more about somatic comorbidities and may experience such comorbidities in a more intense way and, as a consequence, may use health care services more often than non-depressed patients. Besides, it is known that depression in old age is often accompanied by symptoms like fatigue, dizziness, headache, abdominal pain and back pain with unspecific aetiology [44]. These factors might have led to an increased service use of the depressed group. Additionally, depression could make patients mask other somatic illnesses and in consequence delay their treatment [42], resulting in, e.g., prolonged hospital stays or more GP visits as somatic illnesses need a longer time to be revealed. The process of diagnosing somatic comorbidities might thus take longer than for non-depressed patients. For these reasons, patients with depression may have an increased health care utilization. And being more in contact with health care services *ceteris paribus* increases the likelihood of being provided with additional services due to additional comorbidities being detected or treated more intensely.

In multivariate analyses we adjusted incremental costs of depression for comorbidity. Comorbidity was measured by means of the sum of ‘severity points’ of 45 chronic conditions, with the severity of illness rated by the GP. It is possible that patients with depression suffered from comorbidity that is not caught by the created comorbidity score. Moreover, it has been reported that depression-related feelings like hopelessness potentially prevent depressive patients from reporting their somatic symptoms immediately, as these patients are too pessimistic to believe in a successful treatment [45]. This might have affected the employed measurement of comorbidity. Potentially, GPs of the depressed patients underreported their comorbidity as patients might have concealed their severity.

In contrast to the previous explanations that focus on higher resource utilization resulting from depression, causality might be vice versa: Use of care, especially informal care provided by family members, may create a family role imbalance and possible guilt that may manifest as clinical depression. Thus, it might be that it is not depression leading to higher resource utilization but the high use of especially informal care causing depression.

### Comparison with other studies

We found higher total costs for patients with depression compared to non-depressed patients that persisted still after adjustment for functional status and comorbidity. This finding is in a line with results from comparable studies conducted in the USA [41], [42], [44], [46] and in Germany [43], [47], [48], [49]. However, the unadjusted excess costs for depressed patients in our study where much higher (+260%) than reported by other studies for the German health care context (+144% [43], [47]). Yet, both preceding studies from Germany did not include costs of informal care. After excluding costs of informal care in our study, costs for depressed patients still exceeded those for non-depressed by 191% (depressed: €4,323 vs. non-depressed: €2,266). Furthermore, the samples of earlier studies were not restricted to multimorbid patients. Findings for the German health care context have suggested that differences in service utilization between depressed and non-depressed patients increased with increasing comorbidity level [48]. The findings in our study appear to substantiate this because in our sample with a high comorbidity level there were consistently higher differences in costs between depressed and non-depressed patients than reported by previous studies conduced in healthier patients.

### Strengths and limitations

This is the first study comprehensively examining the impact of depression on health care utilization and costs in multimorbid patients. Strengths are that the employed questionnaire recorded in detail the corresponding resource units for all the six most relevant formal health care sectors. Hence, the health care utilization was recorded comprehensively. Additionally, informal care utilization was included and monetarily valued from the societal perspective. For the German health care sector, this is the first cost-of-illness study of depression including informal care.

Limitations arise from the fact that no nursing home residents were recruited. Yet, nursing home residents could be of great relevance in multimorbid elderly. Besides, patients suffering from dementia were excluded. As dementia was an exclusion criterion, the results of our study cannot provide evidence for the multimorbid population with this very common mental illness in old age. An additional limitation could be caused by recall bias due to the six-month period that participants had to recall for the resource utilization.

## Conclusion

In our multimorbid patient sample, higher total costs were found when depression was present. The association persisted after adjusting for comorbidity and functional status. Our study implicates that among multimorbid elderly patients, depression is an important factor for health care utilization and costs. The effect of depression on costs was even greater than found in previous studies with less morbid patients, indicating that it increases with higher multimorbidity.

## References

[1] Luppa M, Sikorski C, Luck T, Ehreke L, Konnopka A, et al (2012) Age- and gender-specific prevalence of depression in latest-life—systematic review and meta-analysis. J Affect Disord 136: 212–221. 21194754 10.1016/j.jad.2010.11.033

[2] Fiske A, Wetherell JL, Gatz M (2009) Depression in older adults. Annu Rev Clin Psychol 5: 363–389. 19327033 10.1146/annurev.clinpsy.032408.153621 PMC2852580

[3] Luppa M, Sikorski C, Motzek T, Konnopka A, König HH, et al (2012) Health service utilization and costs of depressive symptoms in late life - a systematic review. Curr Pharm Des 18: 5936–5957. 22681171 10.2174/138161212803523572

[4] Valderas JM, Starfield B, Sibbald B, Salisbury C, Roland M (2009) Defining comorbidity: implications for understanding health and health services. Ann Fam Med 7: 357–363. 19597174 10.1370/afm.983 PMC2713155

[5] Schäfer I, Hansen H, Schön G, Maier W, Höfels S, et al (2009) The German MultiCare-study: Patterns of multimorbidity in primary health care - protocol of a prospective cohort study. BMC Health Serv Res 9: 145. 19671164 10.1186/1472-6963-9-145 PMC3224741

[6] Lehnert T, Heider D, Leicht H, Heinrich S, Corrieri S, et al (2011) Review: health care utilization and costs of elderly persons with multiple chronic conditions. Med Care Res Rev 68: 387–420. 21813576 10.1177/1077558711399580

[7] Gunn JM, Ayton DR, Densley K, Pallant JF, Chondros P, et al (2012) The association between chronic illness, multimorbidity and depressive symptoms in an Australian primary care cohort. Social psychiatry and psychiatric epidemiology 47: 175–184. 21184214 10.1007/s00127-010-0330-z

[8] Schäfer I, Hansen H, Schön G, Höfels S, Altiner A, et al (2012) The influence of age, gender and socio-economic status on multimorbidity patterns in primary care. first results from the multicare cohort study. BMC Health Serv Res 12: 89. 22471952 10.1186/1472-6963-12-89 PMC3348059

[9] König HH, Born A, Heider D, Matschinger H, Heinrich S, et al (2009) Cost-effectiveness of a primary care model for anxiety disorders. Br J Psychiatry 195: 308–317. 19794198 10.1192/bjp.bp.108.058032

[10] Heinrich S, Luppa M, Matschinger H, Angermeyer MC, Riedel-Heller SG, et al (2008) Service utilization and health-care costs in the advanced elderly. Value Health 11: 611–620. 18179660 10.1111/j.1524-4733.2007.00285.x

[11] Leicht H, Heinrich S, Heider D, Bachmann C, Bickel H, et al (2011) Net costs of dementia by disease stage. Acta Psychiatr Scand 124: 384–395. 21838738 10.1111/j.1600-0447.2011.01741.x

[12] Federal Statistical Office (2010) Verbraucherpreisindizes für Deutschland. Wiesbaden: Federal Statistical Office.

[13] Federal Statistical Office (2008) Kostennachweis der Krankenhäuser. Wiesbaden: Federal Statistical Office.

[14] Federal Statistical Office (2008) Grunddaten der Krankenhäuser. Wiesbaden: Federal Statistical Office.

[15] Deutsche Krankenhausgesellschaft (2009) Bestandsaufnahme zur Krankenhausplanung und Investitionsfinanzierung in den Bundesländern. Berlin: Deutsche Krankenhausgesellschaft.

[16] Deutsche Rentenversicherung Bund (2010) Band 184 - Rehabilitation 2010. Berlin: Deutsche Rentenversicherung Bund

[17] Krauth C, Hessel F, Hansmeier T, Wasem J, Seitz R, et al (2005) Empirische Bewertungsansätze in der gesundheitsökonomischen Evaluation - ein Vorschlag der AG Methoden der gesundheitsökonomischen Evaluation (AG MEG). Gesundheitswesen 67: 736–746. 16235143 10.1055/s-2005-858698

[18] Verband der Ersatzkassen (2001) Vergütungslisten für logopädische/sprachtherapeutische Leistungen. Berlin: Verband der Ersatzkassen (vdek).

[19] Verband der Ersatzkassen (2002) Vergütungsliste für ergotherapeutische Leistungen. Berlin: Verband der Ersatzkassen (vdek).

[20] Verband der Ersatzkassen (2007) Vergütungsliste für podologische Leistungen. Berlin: Verband der Ersatzkassen (vdek).

[21] GKV-Spitzenverband (2007) Festbeträge. Berlin: GKV-Spitzenverband.

[22] Kassenzahnärztliche Bundesvereinigung (2009) Abrechnungshilfe für Festzuschüsse. Köln: Kassenzahnärztliche Bundesvereinigung.

[23] Rote Liste Service GmbH (2008) Rote Liste 2008: Arzneimittelverzeichnis für Deutschland. Frankfurt/Main: Rote Liste Service GmbH.

[24] Federal Statistical Office (2008) Pflegestatistik 2007. Wiesbaden: Statistisches Bundesamt.

[25] Federal Statistical Office (2010) Verdienste und Arbeitskosten - Extraxt (personal communication!). Wiesbaden: Statistisches Bundesamt.

[26] Federal Statistical Office (2009) 2008: Verdienste in Deutschland und Arbeitskosten im EU-Vergleich. Press release no. 179 Wiesbaden: Statistisches Bundesamt.

[27] Yesavage JA, Brink TL, Rose TL, Lum O, Huang V, et al (1982) Development and validation of a geriatric depression screening scale: a preliminary report. J Psychiatr Res 17: 37–49. 7183759 10.1016/0022-3956(82)90033-4

[28] Yesavage JA, Sheikh JI (1986) 9/Geriatric Depression Scale (GDS) Recent Evidence and Development of a Shorter Violence. Clin Gerontologist 5: 165–173.

[29] Allgaier A-K, Kramer D, Mergl R, Fejtkova S, Hegerl U (2011) Validität der Geriatrischen Depressionsskala bei Altenheimbewohnern: Vergleich von GDS-15, GDS-8 und GDS-4. Psychiatrische Praxis 38: 280–286. 21598210 10.1055/s-0030-1266105

[30] Gauggel S, Birkner B (1999) Validität und Reliabilität einer deutschen Version der geriatrischen Depressionsskala (GDS). Zeitschrift für Klinische Psychologie 28: 18–27.

[31] Andersen R, Newman JF (1973) Societal and individual determinants of medical care utilization in the United States. Milbank Mem Fund Q Health Soc 51: 95–124. 4198894

[32] Brauns H, Steinmann S (1999) Educational reform in France, West-Germany and the United Kingdom: updating the CASMIN educational classification. ZUMA Nachrichten 23 (44): 7–44.

[33] Fydrich T, Sommer G, Tydecks S, Brähler E (2009) Fragebogen zur sozialen Unterstützung (F-SozU): Normierung der Kurzform (K-14) Social Support Questionnaire (F-SozU): Standardization of short form (K-14). Zeitschrift für Medizinische Psychologie 18: 43–48.

[34] Sommer G, Fydrich T (1991) Entwicklung und Überprüfung eines Fragebogens zur sozialen Unterstützung (F-SOZU). Diagnostica 37(2): 160–178.

[35] Mahoney FI, Barthel DW (1965) Functional evaluation: the Barthel index. Maryland state medical journal 14: 61–65. 14258950

[36] van den Bussche H, Koller D, Kolonko T, Hansen H, Wegscheider K, et al (2011) Which chronic diseases and disease combinations are specific to multimorbidity in the elderly? Results of a claims data based cross-sectional study in Germany. BMC Public Health 11: 101. 21320345 10.1186/1471-2458-11-101 PMC3050745

[37] Chen J, Shao J (2000) Nearest neighbor imputation for survey data. Journal of Official Statistics 16: 113–132.

[38] van den Berg B, Brouwer WB, Koopmanschap MA (2004) Economic valuation of informal care. An overview of methods and applications. Eur J Health Econ 5: 36–45. 15452763 10.1007/s10198-003-0189-y

[39] Koopmanschap MA, Exel J, Berg B, Brouwer W (2008) An overview of methods and applications to value informal care in economic evaluations of healthcare. Pharmacoeconomics 26: 269–280. 18370563 10.2165/00019053-200826040-00001

[40] Luce BR, Wanning WG, Siegel JE, Lipscomb J (1996) Estimating Costs in Cost-Effectivness Analysis. In: Gold M, Siegel J, Russell L, Weinstein M, editors. Cost-Effectiveness in Health and Medicine. pp. 176–213.

[41] Katon WJ, Lin E, Russo J, Unützer J (2003) Increased medical costs of a population-based sample of depressed elderly patients. Archives of general psychiatry 60: 897. 12963671 10.1001/archpsyc.60.9.897

[42] Unützer J, Patrick DL, Simon G, Grembowski D, Walker E, et al (1997) Depressive symptoms and the cost of health services in HMO patients aged 65 years and older. JAMA: the journal of the American Medical Association 277: 1618–1623. 9168292 10.1001/jama.1997.03540440052032

[43] Luppa M, Heinrich S, Matschinger H, Sandholzer H, Angermeyer MC, et al (2008) Direct costs associated with depression in old age in Germany. Journal of Affective Disorders 105 (1-3): 195–204. 17568683 10.1016/j.jad.2007.05.008

[44] Luber MP, Meyers BS, Williams-Russo PG, Hollenberg JP, DiDomenico TN, et al (2001) Depression and service utilization in elderly primary care patients. The American Journal of Geriatric Psychiatry 9: 169–176. 11316621

[45] Schubert DS, Yokley J, Sloan D, Gottesman H (1995) Impact of the interaction of depression and physical illness on a psychiatric unit's length of stay. General hospital psychiatry 17: 326–334. 8522147 10.1016/0163-8343(95)00065-y

[46] Livingston G, Manela M, Katona C (1997) Cost of community care for older people. Br J Psychiatry 171: 56–59. 9328496 10.1192/bjp.171.1.56

[47] Luppa M, König H-H, Heider D, Leicht H, Motzek T, et al.. (2012) Direct costs associated with depressive symptoms in late life: a 4.5 year prospective study. Int Psychogeriatr. 10.1017/S1041610212001688 23083505

[48] Lacruz ME, Emeny RT, Haefner S, Zimmermann AK, Linkohr B, et al (2011) Relation between depressed mood, somatic comorbidities and health service utilisation in older adults: results from the KORA-Age study. Age ageing 41: 183–190. 22156596 10.1093/ageing/afr162

[49] Busch MA, Neuner B, Aichberger MC, Hapke U, Riedel-Heller SG, et al (2013) [Depressive symptoms and health service utilisation among persons 50 years or older in Germany. A population-based cross-sectional study]. Psychiatr Prax 40: 214–219. 23529658 10.1055/s-0032-1333026

